# Novel Musculocutaneous Flap for Salvage Esophageal Reconstruction

**DOI:** 10.1016/j.atssr.2026.02.013

**Published:** 2026-02-27

**Authors:** Talia G. Meidan, Mohamad K. Abou Chaar, Frank Corl, Carlos A. Puig, Julina Ongkasuwan, Michael J. Klebuc, Andrew T. Huang, N. Eddie Liou, Shawn S. Groth, Shanda H. Blackmon

**Affiliations:** 1 Texas A&M College of Medicine, Bryan, Texas; 2 Department of Surgery, Mayo Clinic, Rochester, Minnesota; 3 Department of Otolaryngology, Baylor College of Medicine, Houston, Texas; 4 Department of Plastic Surgery, Houston Methodist, Houston, Texas; 5 Division of Thoracic Surgery, Department of Surgery, Baylor College of Medicine, Houston, Texas

## Abstract

We present a novel surgical approach for salvage reconstruction of the esophagus in a heavily irradiated field. A tracheoesophageal fistula developed in our patient after laryngectomy and tracheoesophageal puncture for laryngeal cancer. After multiple repairs failed and life-threatening complications, we developed an innovative musculocutaneous flap with extended blood supply from the axillary vessels through a matured saphenous vein loop graft. We term this the "Bring Your Own Blood (BYOB) supply" flap. This case demonstrates the viability of this approach as a last-resort option for esophageal reconstruction in heavily irradiated tissue and highlights important anatomical considerations for patients with vascular anomalies.

Esophageal reconstruction in previously irradiated patients remains highly challenging.^1^ This pathophysiology becomes especially problematic in patients who have received high-dose radiation (>60 Gy), where tissue viability is severely compromised, and the risk of complications increases exponentially.^2^ Tracheoesophageal (TE) fistulas represent a high-morbidity complication.

Standard reconstructive options for esophageal defects include gastric pull-up, colonic interposition, and free jejunal transfer.^1^^,^^3^ The choice depends on various factors, including the level and extent of the defect, prior operations, donor site condition, and comorbidities. Despite advancements in microsurgery, complication rates in irradiated fields can exceed 40%, particularly with fistula or stricture formation.^2^

Alternative strategies, including anterolateral thigh and radial forearm free flaps, have been attempted with mixed success, especially in previously irradiated or anatomically complex patients. Vascular anomalies, such as a right-sided aortic arch with Kommerell diverticulum, further complicate planning and execution.^4^ In these rare, high-stakes scenarios, innovative reconstructive approaches are warranted.

We present a novel "Bring Your Own Blood (BYOB) supply" musculocutaneous flap using a latissimus dorsi free flap with a prematured axillary saphenous vein loop graft, offering a viable salvage option after multiple failed reconstructions in an irradiated field.

A 68-year-old man, who was a former professional athlete, presented with laryngeal squamous cell carcinoma. His medical history was significant for 25 pack-year tobacco use. He underwent laryngectomy with tracheoesophageal (TE) puncture for voice restoration and received 70 G of radiation therapy to the neck.^5^

A TE fistula with aspiration subsequently developed. Initial attempts at repair with local rotational pectoralis muscle flaps by the otolaryngology service failed, likely due to radiation-induced tissue damage. Owing to continued aspiration and malnutrition, combined with the patient's refusal of a feeding tube, the gastroenterology service placed an esophageal stent to cover the fistula.

When the stent migrated after a few days, our surgical team was consulted. A computed tomography (CT) scan performed after the stent was tacked in position revealed a previously undiagnosed right-sided aortic arch with a Kommerell diverticulum. The patient left the hospital against medical advice, even after the identification of this vascular anomaly, which posed a risk of aortic erosion.

Despite these warnings, the patient did not return for 3 weeks and later presented with massive hematemesis. Emergent angiography confirmed stent erosion into the aorta. The patient was stabilized with a Sengstaken-Blakemore tube, followed by endovascular stenting of the aorta. Subsequently, the Kommerell diverticulum was oversewn through a thoracotomy, and the esophagus was diverted.

A supercharged jejunal interposition using the left internal mammary artery and vein was attempted for esophageal reconstruction (Figure 1). However, the proximal anastomosis failed due to the severely irradiated field. At this point, with conventional reconstructive options exhausted, we devised a novel approach.

**Figure 1:**
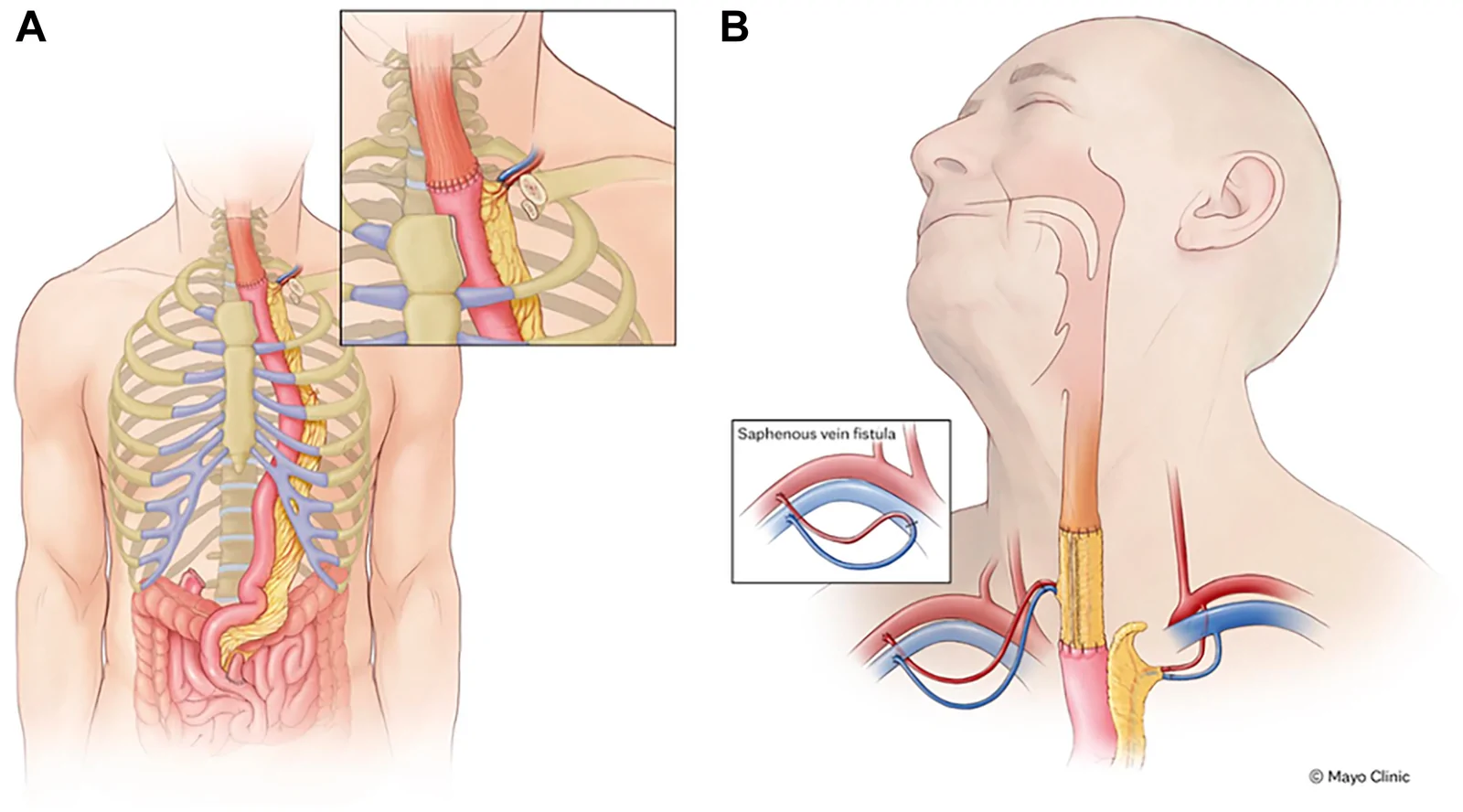
(A) Supercharged jejunum. (From: Blackmon SH. Long-segment, supercharged pedicled jejunal interposition for esophageal replacement: how I teach it. *Ann Thorac Surg*. 2018;105:345-350; used with permission of Mayo Foundation for Medical Education and Research, all rights reserved.) (B) Saphenous vein loop graft. The small window is a close-up of the left internal mammary artery and vein connected to the jejunal mesenteris artery and vein, respectively. (Used with permission of Mayo Foundation for Medical Education and Research, all rights reserved.)

We accomplished the BYOB flap technique by creating an extended blood supply for a right latissimus dorsi musculocutaneous flap by:

1. Constructing a saphenous vein loop graft (from the right axillary artery to the axillary vein) (Figure 1)
2. Allowing this loop to mature for 2 weeks, similar to an arteriovenous fistula for dialysis access
3. Harvesting the latissimus dorsi musculocutaneous flap with its native thoracodorsal vessels
4. Connecting the free flap's blood supply to the prematured saphenous vein loop, which was divided in half for arterial and venous supply
5. Using a linear stapler to create the tube forming the myocutaneous component to create a neoesophagus, we were able to place the tubularized (skin on the inside of the tube) flap with ample muscle (seen in darker peach color in illustration) to create a neoconduit, for a cross-section of the neck depicting this reconstruction, please refer to Figure 2

**Figure 2:**
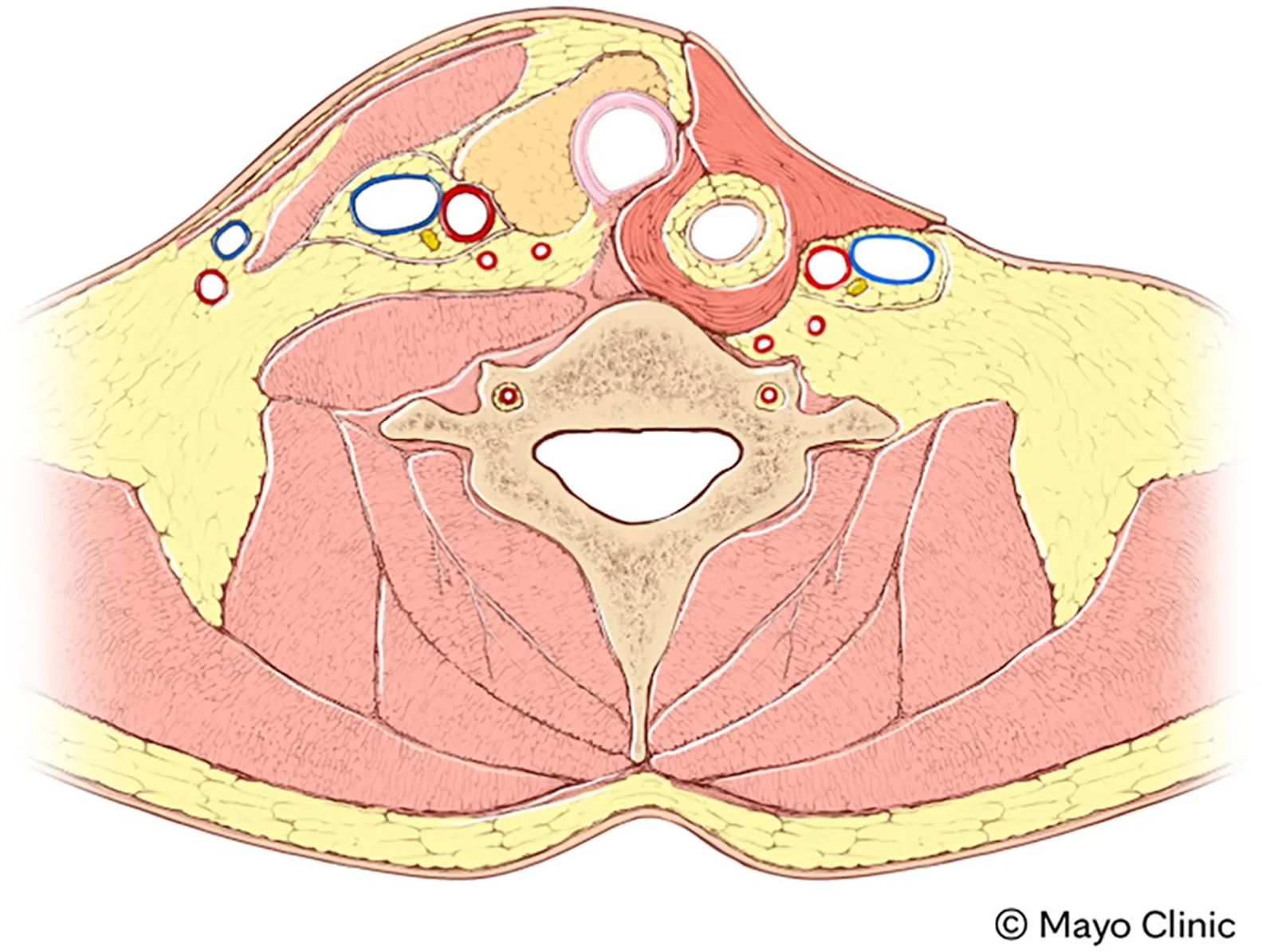
Myocutaneous free flap. (Used with permission of Mayo Foundation for Medical Education and Research, all rights reserved.)
6. Performing skin grafting over the exposed muscle surface (Figure 3)

**Figure 3:**
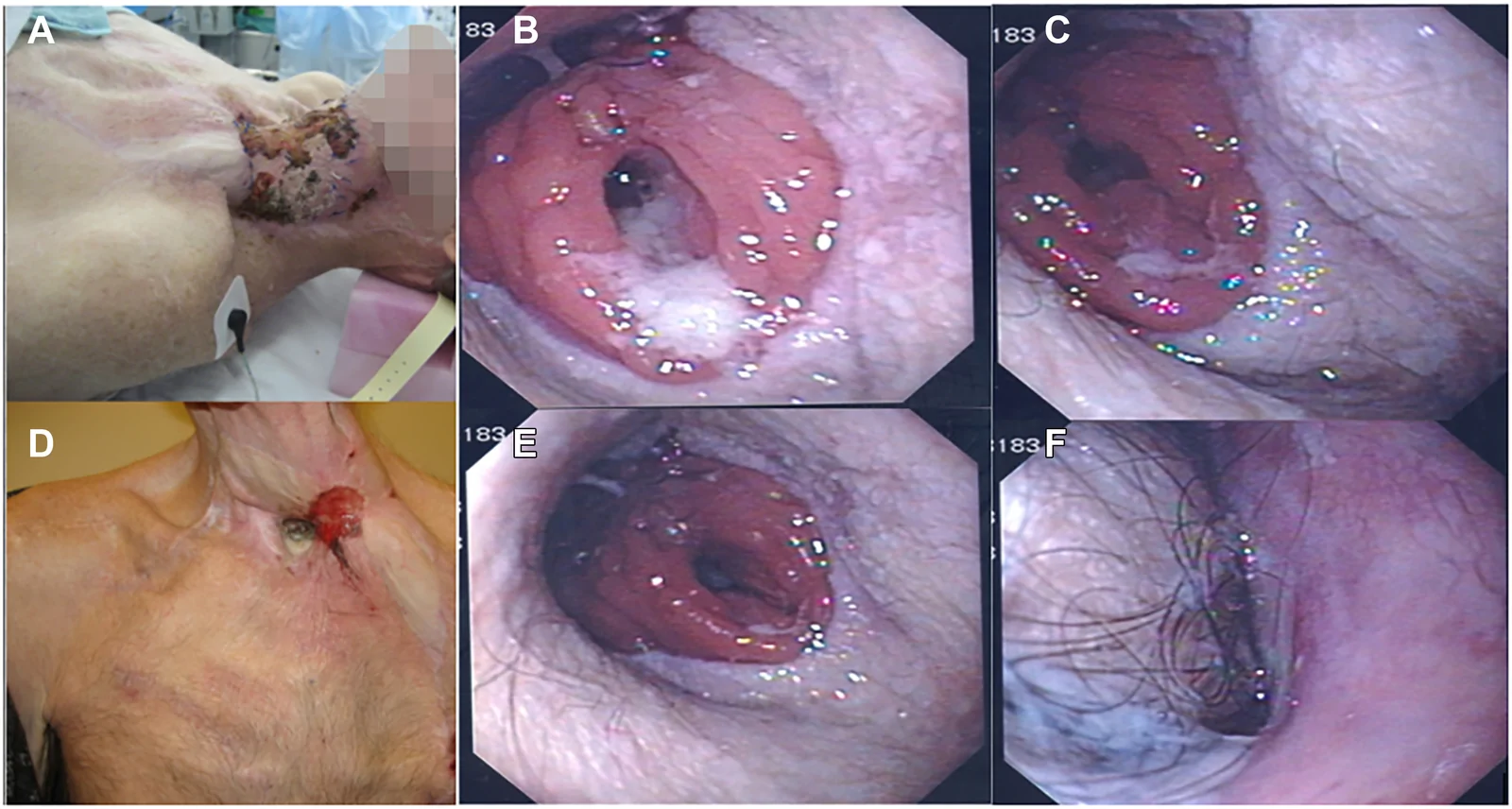
(A) Top left, skin graft partial coverage over the myocutaneous flap used to interpose between the native esophagus and the jejunal conduit at the level of the neck. (B) Top central image, endoscopic view of the distal skin tube healed anastomosis to the jejunal interposition with lumen in the center. (C) Top right, endoscopic view of the distal skin tube healed anastomosis to the jejunal interposition with lumen on the left of the screen and hair present within the skin tube. (D) Bottom right, patient appearance upon follow-up in clinic with no leak, tolerating an oral diet, and continued healing of the skin adjacent to the mediastinal tracheostomy. Incisions from the vein loop can be seen on the patient's right clavicular region. (E) Bottom center, endoscopic view of the middle portion of the lumen of the skin tube demonstrating a healed anastomosis to the jejunal interposition with lumen in the center. (F) Bottom right, endoscopic view of the proximal skin tube with hair covering the inner lining of the neoconduit of skin.

Postoperative endoscopy revealed a patent neoesophagus with the unique finding of hair-bearing skin visible on the luminal surface—a "hairy esophagus" (Figure 3).

The patient achieved successful oral nutrition for ∼1 year after the procedure. Despite what would be termed a complete success; unfortunately, a urinary tract infection developed, and consistent with his previously established advance directives, he declined antibiotic treatment and died.^5^

## Comment

The BYOB flap offers a novel solution for esophageal reconstruction in the setting of radiation-impaired tissue, where conventional techniques often fail due to compromised vascularity.^1^ By creating a prematured arteriovenous loop with saphenous vein graft between the axillary artery and vein, we effectively bypassed the compromised vasculature in the radiation field, providing robust blood supply to the latissimus dorsi musculocutaneous flap. This approach shares conceptual similarities with the supercharged jejunal flap technique described by Blackmon and colleagues,^3^ which demonstrated excellent functional outcomes in total esophageal reconstruction.

This case underscores the importance of preoperative vascular evaluation. The patient’s undiagnosed right-sided aortic arch with Kommerell diverticulum complicated management and led to a life-threatening stent erosion. Careful vascular assessment may prevent such events.^4^

Despite a technically successful supercharged jejunal interposition, radiation damage contributed to anastomotic failure, consistent with prior findings that even optimal techniques carry high fistula and stricture rates in these fields.^6^^,^^7^ The BYOB flap succeeded where others failed, demonstrating its utility as a last-resort option.

Interestingly, the “hairy esophagus” observed reflects the expected use of skin-lined flaps and did not compromise function.^8^ Multidisciplinary collaboration was critical, involving otolaryngology, thoracic, vascular, gastroenterology, and reconstructive surgery to manage this complex patient.^1^

The BYOB flap should be considered a last-resort option for esophageal reconstruction when conventional methods fail. It is particularly suited for patients with heavily irradiated tissues with compromised vasculature. The BYOB flap can be an option for pharyngeal, esophageal, tracheal, or bronchial reconstruction.

The novel BYOB flap represents a viable salvage option for esophageal reconstruction in heavily irradiated tissues where conventional approaches have failed. By creating an extended blood supply through a prematured saphenous vein loop between the axillary artery and vein, we successfully reconstructed the esophagus in a patient with multiple failed prior attempts. This technique bypasses the compromised vasculature in radiation fields, enhancing flap survival.

This case highlights important considerations for reconstructive surgeons: the importance of preoperative vascular assessment, the value of multidisciplinary collaboration in complex reconstruction, and the role of innovative vascular techniques in addressing challenging reconstructive problems in the irradiated head and neck.

Although the BYOB flap should be considered a last-resort option for esophageal reconstruction, this approach provides a valuable addition to the reconstructive surgeon's repertoire for treating complex esophageal defects.
